# Teriflunomide Inhibits JCPyV Infection and Spread in Glial Cells and Choroid Plexus Epithelial Cells

**DOI:** 10.3390/ijms22189809

**Published:** 2021-09-10

**Authors:** Bethany A. O’Hara, Gretchen V. Gee, Sheila A. Haley, Jenna Morris-Love, Charlotte Nyblade, Chris Nieves, Barbara A. Hanson, Xin Dang, Timothy J. Turner, Jeffrey M. Chavin, Alex Lublin, Igor J. Koralnik, Walter J. Atwood

**Affiliations:** 1 Department of Molecular Biology, Cell Biology and Biochemistry, Brown University, Providence, RI 02903, USA; bethany_ohara@brown.edu (B.A.O.); sheila_haley@brown.edu (S.A.H.); jenna_morris-love@brown.edu (J.M.-L.); charlotte_nyblade@brown.edu (C.N.); chris_nieves@brown.edu (C.N.); 2 MassBiologics, University of Massachusetts Medical School, Worcester, MA 01601, USA; Gretchen.Gee@umassmed.edu; 3 Davee Department of Neurology, Northwestern University Feinberg School of Medicine, Chicago, IL 60007, USA; barbara.hanson@northwestern.edu (B.A.H.); xin.dang@northwestern.edu (X.D.); igor.koralnik@northwestern.edu (I.J.K.); 4 Sanofi, Cambridge, MA 02114, USA; Timothy.Turner@sanofi.com (T.J.T.); jeffrey.chavin@sanofi.com (J.M.C.); alex.lublin@sanofi.com (A.L.)

**Keywords:** multiple sclerosis, demyelination, glia, autoimmunity, neuroinflammation, choroid plexus, extracellular vesicle, polyomavirus

## Abstract

Several classes of immunomodulators are used for treating relapsing-remitting multiple sclerosis (RRMS). Most of these disease-modifying therapies, except teriflunomide, carry the risk of progressive multifocal leukoencephalopathy (PML), a severely debilitating, often fatal virus-induced demyelinating disease. Because teriflunomide has been shown to have antiviral activity against DNA viruses, we investigated whether treatment of cells with teriflunomide inhibits infection and spread of JC polyomavirus (JCPyV), the causative agent of PML. Treatment of choroid plexus epithelial cells and astrocytes with teriflunomide reduced JCPyV infection and spread. We also used droplet digital PCR to quantify JCPyV DNA associated with extracellular vesicles isolated from RRMS patients. We detected JCPyV DNA in all patients with confirmed PML diagnosis (*n* = 2), and in six natalizumab-treated (*n* = 12), two teriflunomide-treated (*n* = 7), and two nonimmunomodulated (*n* = 2) patients. Of the 21 patients, 12 (57%) had detectable JCPyV in either plasma or serum. CSF was uniformly negative for JCPyV. Isolation of extracellular vesicles did not increase the level of detection of JCPyV DNA versus bulk unprocessed biofluid. Overall, our study demonstrated an effect of teriflunomide inhibiting JCPyV infection and spread in glial and choroid plexus epithelial cells. Larger studies using patient samples are needed to correlate these in vitro findings with patient data.

## 1. Introduction

The majority of human polyomaviruses cause long-term asymptomatic persistent infections in their host [1]. Several, however, are associated with disease in immunocompromised or immunomodulated patients (JC polyomavirus [JCPyV], BK polyomavirus [BKPyV], Trichodysplasia spinulosa polyomavirus [TSPyV]) or with the development of cancer (Merkel cell polyomavirus [MCPyV]) [1]. JCPyV is the only member of the family to cause neurologic disease [2,3]. In the context of prolonged immunosuppression or prolonged treatment with powerful immunomodulators, JCPyV can give rise to an often fatal demyelinating disease known as progressive multifocal leukoencephalopathy (PML) [2,4]. Several immunomodulators used to treat patients with multiple sclerosis (MS) have black box warnings about the risk of developing PML [5]. Natalizumab was the first of these newer drugs to be shown to increase the risk of PML in patients with MS and other autoimmune patients and remains the drug most associated with the development of PML [6,7,8]. Unlike other drugs prescribed for relapsing-remitting MS (RRMS), teriflunomide is not associated with the development of PML [9]. Several studies have shown that teriflunomide can also inhibit the replication of DNA viruses, and one study showed it could reduce infection of cells by the related BKPyV [10,11]. In this study, we sought to determine whether teriflunomide could inhibit the infection of cells by JCPyV. We found that nontoxic doses of teriflunomide inhibited initial infection and spread of JCPyV in known target cells in vitro. The ability to reduce infection was correlated with teriflunomide’s cytostatic effect. Because teriflunomide is known to inhibit de novo pyrimidine synthesis, we attempted to rescue the inhibition by supplementing cells with uridine or the metabolic intermediate orotic acid [12]. Neither was capable of rescuing JCPyV infection in teriflunomide-treated cells, indicating that other mechanisms were responsible for teriflunomide’s ability to reduce viral infection. We also quantified JCPyV DNA in extracellular vesicles (EV) isolated from cerebrospinal fluid (CSF), serum, and plasma of patients with RRMS whose treatments included teriflunomide or natalizumab. As controls, we obtained CSF and plasma from two patients with well-documented PML. We found low copy numbers of JCPyV DNA in serum and plasma of several of the non-PML RRMS patient samples. Plasma samples were more likely to be positive than serum samples, and CSF samples were uniformly negative in non-PML patients. Isolating the EV from these biofluids did not result in increased sensitivity of detecting JCPyV DNA in these samples.

## 2. Results

### 2.1. Cytotoxicity and Cytostatic Effect of Teriflunomide

**Figure 1:**
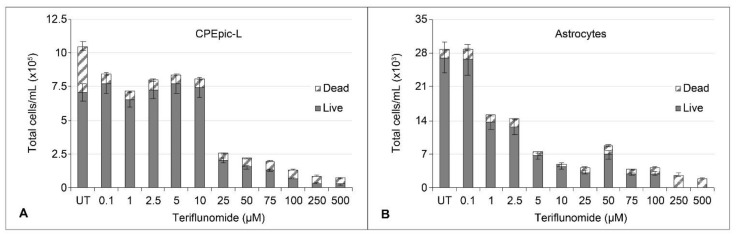
Live/dead cell counts of JCPyV-infected, teriflunomide-treated CPEpic-L (**A**), and primary astrocytes (**B**). Cells were pretreated, infected, and maintained in teriflunomide for 5 days. On Day 5, cells were collected using Cellstripper, stained with propidium iodide, and analyzed by flow cytometry for total live/dead cell counts. Volume-matched vehicle controls were run for each sample. There was a dose-dependent decrease in total cell number for all cell lines, and viability was maintained at all but the highest doses of teriflunomide. Bars represent the average of each sample count in triplicate. Error bars represent standard deviation. CPEpic-L = choroid plexus epithelial cells; JCPyV = JC polyomavirus.

Propidium iodide staining and flow cytometry were used to distinguish live from dead cells following treatment of a choroid plexus epithelial cell line (CPEpic-L) or of primary human astrocytes [13,14]. The hTert immortalized CPEpic-L showed a marked reduction in the total number of cells above 10 µM but cells remained viable up to 100 µM (Figure 1A). Primary astrocytes showed marked reduction in the total number of cells above 0.1 µM but cells remained viable up to 100 µM (Figure 1B).

### 2.2. Teriflunomide Inhibits Initial Infection and Virus Spread over Time

**Figure 2:**
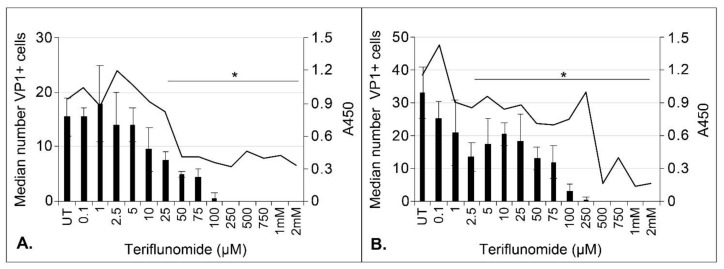
The ability of teriflunomide to reduce JCPyV infection correlates with teriflunomide’s cytostatic effect in CPEpic-L and primary astrocytes. CPEpic-L (**A**) and primary astrocytes (**B**) were pretreated with teriflunomide for 1 h, infected, and maintained in the presence of teriflunomide for 5 days. Cells were fixed in 100% methanol and analyzed by indirect immunofluorescence analysis of VP1. The median number of VP1-positive cells (left axis) is plotted against the A450 (right axis) to show the relationship between decreased infection and the cytostatic effect of teriflunomide. Error bars indicate the IQR of each sample. There is a significant reduction in infection in CPEpic-L cells at 25 µM and above and in astrocytes at 2.5 µM and above. * = *p* < 0.05, Student’s *t*-test; UT = untreated; VP1 = viral protein 1; IQR = interquartile range.

The ability of teriflunomide to inhibit initial infection of CPEpic-L and primary astrocytes was tested. Cells were pretreated with increasing doses of teriflunomide for 1 h and then challenged with cesium chloride–purified JCPyV (multiplicity of infection [MOI] = 100 ffu/cell). At 5 days post infection, cells were fixed in methanol and infection was scored by counting viral protein 1 (VP)1-positive cells using Elements High Content imaging software (Nikon Inc., Chicago, IL USA). The metabolic activity of the cells was measured in parallel using the CellTiter 96 Aqueous One Solution Cell Proliferation Assay (MTS) (Promega, Madison, WI USA). The decline in JCPyV-infected cells closely mirrored the cytostatic effect of the drug in CPEpic-L and primary astrocytes (Figure 2).

**Figure 3:**
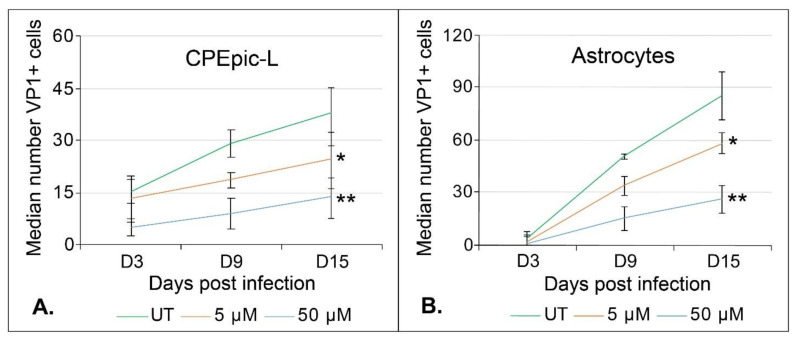
Teriflunomide inhibits the spread of JCPyV in CPEpic-L and primary astrocytes. CPEpic-L (**A**) and primary astrocytes (**B**) were infected with JCPyV and treated with volume-matched vehicle control (DMSO) or with teriflunomide 24 h post infection. The cells were maintained for 15 days with weekly changes of media that contained vehicle or teriflunomide. Infectivity was measured by staining the cells for indirect immunofluorescence analysis of VP1. The median value of VP1+ cells is reported. Teriflunomide significantly reduced JCPyV spread in both CPEpic-L and in primary astrocytes at 9 and 15 days post infection when treated with 50 µM and at 15 days post infection when treated with 5 µM teriflunomide. (* *p* < 0.05, Student’s *t*-test, ** *p* < 0.01, Student’s *t*-test). Error bars indicate the interquartile range (IQR) of each treatment.

We also assessed the ability of lower doses of teriflunomide to reduce virus growth over a 15-day multicycle growth assay. Cells were infected with virus first and then treated with vehicle control or teriflunomide 24 h post infection. The cells were maintained for 15 days with new media and treatment added weekly. Teriflunomide at 5 µM and 50 µM concentrations significantly reduced virus spread in both CPEpic-L and primary astrocytes (Figure 3).

### 2.3. Inhibitory Effect of Teriflunomide on Viral Infection Cannot Be Rescued by Supplementing Cells with Uridine or Orotic Acid

**Figure 4:**
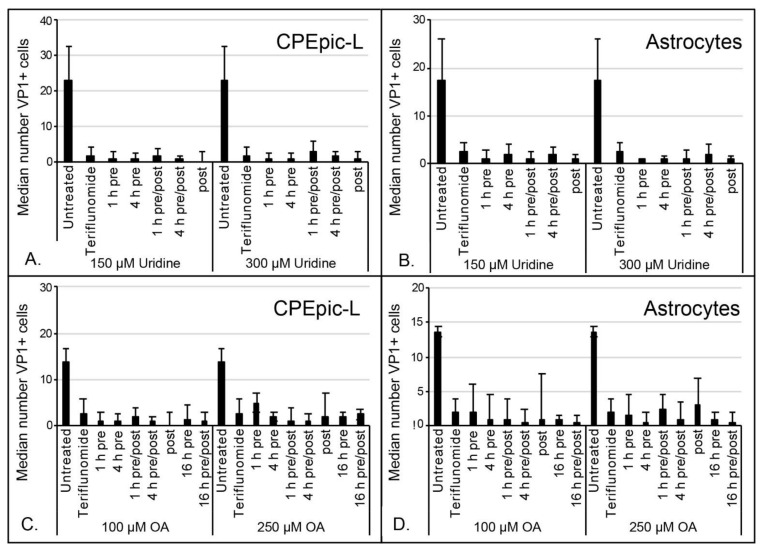
Uridine or orotic acid failed to rescue JCPyV infection in teriflunomide-treated cells. CPEpic-L (**A**,**C**) and primary astrocytes (**B**,**D**) were infected and untreated or treated with teriflunomide, and treated with uridine (**A**,**B**) or orotic acid (**C**,**D**). Cells were treated with uridine for 1 h or 4 h prior to teriflunomide and maintained in teriflunomide only; or treated with uridine for 1 h or 4 h and maintained in both teriflunomide and uridine; or maintained in both uridine and teriflunomide without uridine pretreatment. Cells were treated with orotic acid for 1 h, 4 h, or 16 h prior to teriflunomide and maintained in teriflunomide only; or treated with orotic acid for 1 h, 4 h, or 16 h and maintained in both teriflunomide and orotic acid; or maintained in both orotic acid and teriflunomide without orotic acid pretreatment. All conditions were carried out in triplicate at high and low concentrations of uridine (300 µM and 150 µM, respectively) or orotic acid (250 µM and 100 µM, respectively) and using the previously established highest tolerable doses of teriflunomide as appropriate per cell type. Neither uridine nor orotic acid was able to rescue JCPyV infection under any of the tested conditions; there are no significant differences between the teriflunomide only treated samples and the orotic acid or uridine treated samples (*p* > 0.05 for all conditions containing uridine and orotic acid; Student’s *t*-test). Error bars indicate the interquartile range of each treatment. OA = orotic acid; VP1 = viral protein 1.

Teriflunomide is known to reduce lymphoid cell proliferation by inhibiting dihydroorotate dehydrogenase, which is involved in de novo pyrimidine synthesis [12]. To determine whether this pathway was critical during in vitro viral infection of CPEpic-L and primary astrocytes, we supplemented teriflunomide-treated cells with 150 µM and 300 µM uridine or with 100 µM and 250 µM orotic acid, a metabolic intermediate downstream of dihydroorotate dehydrogenase. Supplementation with either compound was unable to rescue infection in teriflunomide-treated CPEpic-L or primary astrocytes (Figure 4), indicating either other mechanisms were responsible for teriflunomide’s ability to reduce viral infection or that inadequate levels of drug reached the cytoplasm.

### 2.4. Capturing EV from CSF, Plasma, and Serum

**Figure 5:**
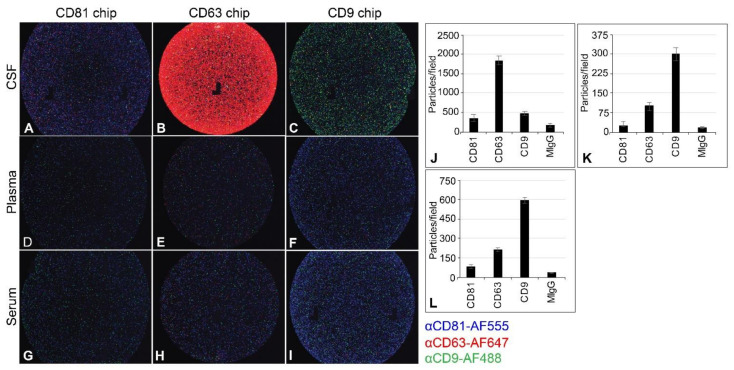
Exoview analysis of tetraspanin presentation on EV derived from CSF, plasma, and serum. Unlabeled EV were captured on CD9, CD63, or CD81 antibody chips. Bound EV were stained with αCD9-AF488 (green), αCD81-AF555 (blue), and αCD63-AF647 (red) (**A**–**I**). The intensity of each fluorescent antibody signal reflects the relative abundance of each tetraspanin. CD63 is the predominant EV marker in CSF (**B**,**J**). CD9 is the predominant EV marker in plasma (**F**,**K**) and serum (**I**,**L**). Average particle counts per 150-µM field of view for each captured tetraspanin before staining are shown for CSF (**J**), plasma (**K**), and serum (**L**). As expected, there are differences in tetraspanin expression between sample types. Error bars represent standard deviation. CSF = cerebrospinal fluid; EV = extracellular vesicles; MIgG = mouse immunoglobulin G.

Because of the inhibitory effect of teriflunomide in vitro, we sought to determine whether patients treated with teriflunomide had lower viral loads than those who were untreated or treated with natalizumab, and whether isolation of EV—known to harbor JCPyV—would increase the sensitivity of detecting JCPyV genomes in patient samples. We developed and validated an assay for isolating EV from small volumes of control biofuid spiked with known quantities of EV isolated from JCPyV-infected cells. Healthy donor CSF, plasma, and serum were obtained from a commercial biobank (Innovative Research, Novi, WI, USA). We found that a combination of size-exclusion chromatography and ultrafiltration gave the best results based on size, purity, and expression of EV-associated markers CD81, CD63, and CD9. Exoview analysis (NanoView Biosciences, Boston, MA, USA) confirmed that we could capture significant numbers of EV from each biofluid and also confirmed the known distribution of markers expressed on EV from each biofluid (Figure 5).

### 2.5. Workflow and Standard Curves for Droplet Digital PCR (ddPCR) Analysis of EV-Associated JCPyV DNA

**Figure 6:**
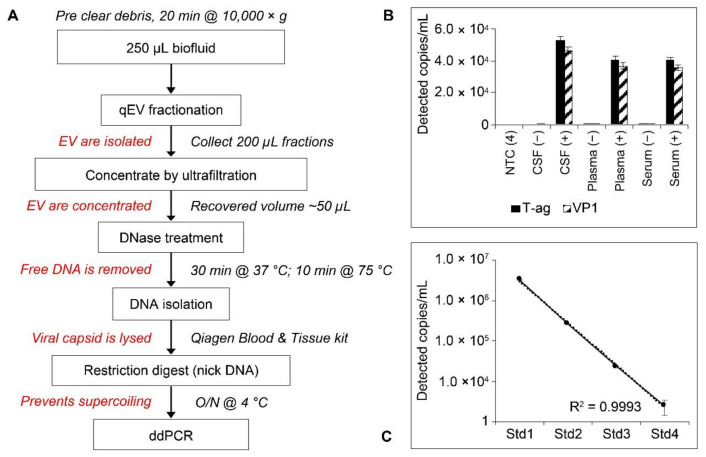
(**A**) Workflow and absolute quantification of JCPyV in plasma-, serum-, and CSF-derived EV. Healthy donor control samples were either spiked with infectious EV or PBS control. EV were isolated and concentrated, and viral DNA was extracted. Following digestion, ddPCR was performed to determine viral genome number. (**B**) There was consensus both within and between biofluid types for detected viral copies. Standard curve of Mad1-pBR322 plasmid DNA by ddPCR. (**C**) Detected genome equivalent count matched the known input for each standard and was reproducible. R^2^ = 0.9993. ddPCR = droplet digital PCR; NTC = no-template control; O/N = overnight; PBS = phosphate-buffered saline; T-ag = T-antigen. Error bars represent the standard deviation.

A multistep protocol was used to accurately quantify EV-associated virus (Figure 6). This protocol lyses EV carrying JCPyV as cargo and releases the encapsidated DNA, which is then quantified for the absolute number of genome equivalents per µL of input sample. We established protocols for the detection of JCPyV with Taqman primer/probe sets using absolute quantification of viral genomes by ddPCR. Because of the unknown viral load status of the RRMS patient library obtained from Innovative Research, ddPCR was chosen as the most sensitive method of detection; the lower limit of detection is a single genome copy per well. To ensure accuracy and specificity, probe sets across two viral targets were designed (the T-antigen region and the viral capsid protein-encoding region VP1). To validate the specificity of our assay, we performed a standard curve using known genome equivalents. Standards were generated by making 10-fold dilutions of the JCPyV plasmid Mad1-pBR322 in sterile water. The DNA concentration of each dilution was checked in triplicate by Qubit DNA high sensitivity (Invitrogen, Waltham, MA USA), and copy number was calculated using the amount of DNA and template length. The detected concentration of each standard (in genomes/µL) very closely matched our known input amounts, demonstrating that our assay was both sensitive and accurate (Figure 6). We next spiked each biofluid (plasma, serum, and CSF) with 5 µL of EV isolated from JCPyV-infected SVGA glial cells. The EV were then isolated using size-exclusion chromatography mini-columns followed by ultrafiltration of the viral-rich fractions using Amicon Ultra-4 columns (Merck-Millipore, Burlington, MA, USA). Purified and concentrated EV were processed according to the workflow diagram and analyzed by ddPCR for JCPyV T-antigen and VP1. A representative sample of each biofluid was also processed without the addition of EV. Samples were processed by ddPCR using the standard protocol for probes as described in methods Section 4.9. A droplet stabilization step for 4 h at 12 °C was added prior to droplet analysis. Data were processed using BioRad QuantaSoft software (Bio-Rad Laboratories, Hercules, CA, USA). Probe sets across multiple viral targets detected approximately the same number of genome copies per well within each sample type (Figure 6). There was also consensus between sample types for detected genome concentration.

### 2.6. Analysis of JCPyV DNA in Patient Samples

**Figure 7:**
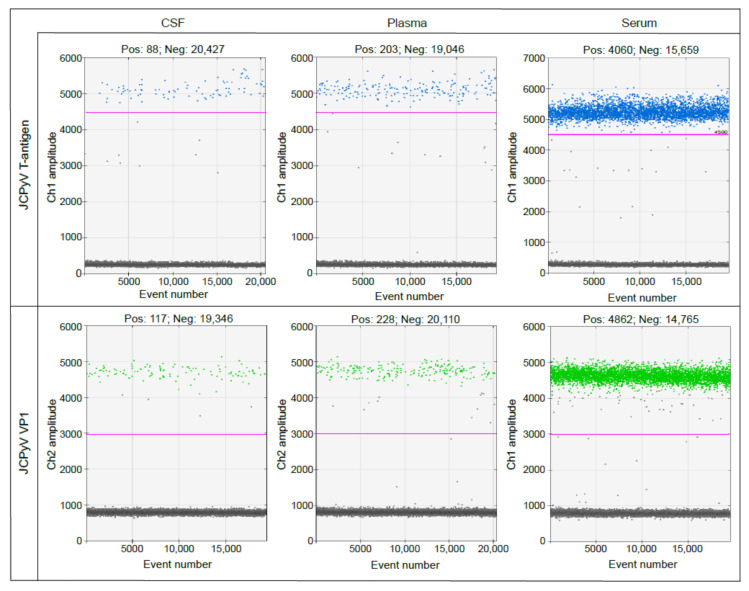
Examples of droplet count and thresholding used in the analysis. Controls were generated for each biofluid (plasma, serum, and CSF) by spiking 250 µL healthy donor fluid with 5 μL JCPyV-positive EV. EV were isolated using size-exclusion chromatography mini-columns (Izon, Christchurch, NZ) followed by ultrafiltration using Amicon Ultra-4 columns (Merck-Millipore, Burlington, MA, USA). Purified and concentrated EV were processed according to the workflow diagram and analyzed by ddPCR for JCPyV T-antigen and VP1 expression. The amplitude of each positive control was used to determine the threshold setting, shown as a pink line, for each probe set.

RRMS patient samples obtained from Innovative Research (Novi, WI, USA) were processed using the EV workflow (Figure 6) and directly extracted for ddPCR analysis to determine viral loads in the unprocessed samples. All samples were processed by ddPCR using the protocol described in Methods. A droplet stabilization step for 4 h at 12 °C was added prior to droplet analysis. Data were processed using BioRad QuantaSoft software (Bio-Rad Laboratories, Hercules, CA, USA). The parameters we set for sample positivity included that samples must have at least two fluorescent droplets, the droplets must be at the expected amplitude (expected amplitude for T-antigen probe set, 5000–6000; for VP1 probe set, 4000–5000), and there must be consensus between the two probe sets. Examples of droplet count and thresholding used in the analysis are shown in Figure 7.

**Figure 8:**
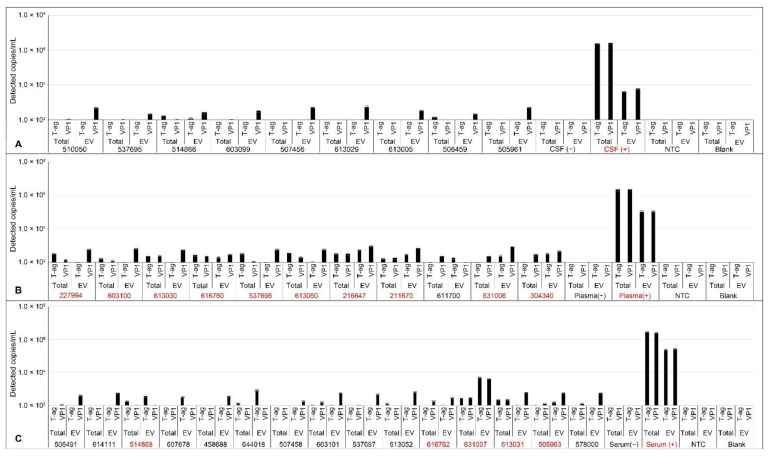
Individual ddPCR results for detection of JCPyV in CSF (**A**), plasma (**B**), and serum (**C**). EV were isolated using size-exclusion chromatography mini columns followed by ultrafiltration using Amicon Ultra-4 columns. Purified and concentrated EV were processed according to the workflow diagram and analyzed by ddPCR for JCPyV genomes. Total samples were DNA extracted, digested, and similarly analyzed. The y-axis represents 120 copies/mL and is the equivalent of two positive drops at the correct amplitude. JCPyV-positive samples are shown in red. Error bars represent the standard error.

**Figure 9:**
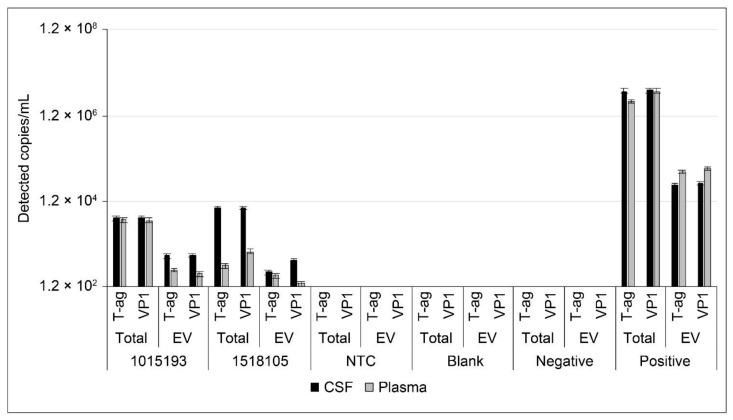
Individual ddPCR results for detection of JCPyV in CSF- and plasma-derived EV from patients with diagnosed PML. EV were isolated using size-exclusion chromatography mini columns followed by ultrafiltration using Amicon Ultra-4 columns. Purified and concentrated EV were processed according to the workflow diagram and analyzed by ddPCR for JCPyV genomes. Total samples were DNA extracted, digested, and similarly analyzed. The y-axis represents 120 copies/mL and is the equivalent of two positive drops at the correct amplitude. All of these samples are considered JCPyV positive. PML = progressive multifocal leukoencephalopathy. Error bars represent the standard error.

Twenty-one patients with RRMS and two with PML were included in this study (Table 1 and Supplementary Table S1). Of the 21, 12 patients (57%) had detectable JCPyV in either plasma or serum. CSF from patients with RRMS in this study was uniformly negative for JCPyV. Patient information was used to group patients into four categories: teriflunomide treated (*n* = 7), natalizumab treated (*n* = 12), nonimmunomodulated (*n* = 2), and patients with human immunodeficiency virus (HIV)-associated PML (HIV-PML; *n* = 2). Three of seven plasma EV fraction samples and six of seven total plasma samples from natalizumab patients were low-level positive. Subclinical reactivation of JCPyV following natalizumab treatment was previously reported and may be the cause of these results [7]. One of six total serum samples and zero of six serum EV samples from teriflunomide patients were positive. One patient, a nonimmunomodulated 76-year-old male, had significant amounts of detectable JCPyV in EV derived from serum and a low level of detection in plasma EV (Table 1, Figure 8, and Supplementary Table S1). Spinal fluid from the same patient was negative for JCPyV (Table 1, Figure 8, and Supplementary Table S1). The CSF and plasma from both PML patients in the study were positive for JCPyV (Figure 9). That JCPyV was detected in any patient highlights the importance of monitoring patients with MS as a potential early indicator for the development of PML using sensitive methods such as ddPCR.

## 3. Discussion

The JCPyV-induced disease PML was once an extremely rare event that occurred in the context of lymphoid cancers such as leukemia and Hodgkin’s disease [15,16]. In the early 1980s, PML emerged as a fatal complication of HIV infection and in 2005 it emerged as a fatal complication in patients with autoimmune disease being treated with powerful new immunomodulatory drugs [17,18,19,20,21,22,23]. PML has been largely controlled in the HIV/AIDS population by antiretrovirals that keep HIV in check and prevent immunosuppression. There has also been significant progress in preventing the development of PML in patients being treated with natalizumab by careful and frequent screening for JCPyV reactivation and by the implementation of extended-interval dosing schedules that may preserve enough immune function to reduce the likelihood of PML [24,25,26,27]. Since 2005, over a dozen different immunomodulatory drugs have been developed to treat autoimmune disease and the majority have been associated with PML [4]. Teriflunomide, whose mechanism of action appears restricted to reducing the proliferation of lymphoid cells, has not yet been associated with PML [9]. Because teriflunomide has been shown to inhibit DNA viruses that require cell proliferation, we evaluated whether the lack of PML in teriflunomide-treated patients might be attributed to its antiviral activity. We examined this in CPEpic-L and in primary human astrocytes. The rationale for choosing CPEpic-L is that the choroid plexus has been newly identified as a tissue susceptible to JCPyV infection both in vivo and in vitro [13,14,28]. Infection of the choroid plexus epithelium at the blood-CSF barrier may modulate viral invasion of brain parenchyma to disseminate infection. Moreover, choroid plexus epithelial cells play an important role in communicating between the blood and the brain using EV [29]. Several groups have shown that EV harbor JCPyV genomes both in vivo and in vitro and contribute significantly to virus invasion of naïve cells [30,31]. EV are also capable of carrying mutant viruses that can only infect cells when associated with these vesicles [13,14,32]. Primary astrocytes were chosen as they more closely reflect normal human astrocytes than the SV40 T-antigen–transformed SVGA cell line, the cell line more often used for in vitro work with JCPyV. Our data clearly show that teriflunomide reduces initial infection of CPEpic-L and primary astrocytes by JCPyV, and the ability of teriflunomide to reduce infection is directly related to its ability to suppress cell proliferation. Because teriflunomide is known to suppress the proliferation of lymphocytes by inhibiting the enzyme dihydroorotate dehydrogenase, we evaluated whether the inhibitory effect of the drug could be rescued by supplying uridine or the metabolic intermediate orotic acid [12]. Neither compound could overcome the antiviral effect of teriflunomide, suggesting the mechanism does not involve dihydroorotate dehydrogenase in these cell types, as has been previously reported [10].

In an attempt to correlate our in vitro findings with patient data, we obtained CSF, plasma, and serum from patients with RRMS whose treatments included teriflunomide and natalizumab. We determined conditions for isolating EV from small volumes of these biofluids and amplifying viral genomes by ddPCR. We examined both bulk biofluid and the EV fractions to determine whether isolation of the EV fraction would increase the sensitivity of detecting viral genomes in these patient samples. In the two patients with PML, both CSF and plasma were strongly positive for JCPyV genomes when either the bulk biofluid or the EV fraction was tested. Consistent with other reports of viral genomes in EV, only a fraction of the total genome copy numbers were found to be associated with the EV [30,31]. It is unclear whether this is because there were fewer viral genomes in the EV compared with the total or whether this represents a loss of EV during processing, which included size-exclusion chromatography and ultrafiltration. We are currently designing experiments to distinguish between these two possibilities. In natalizumab-treated patients without PML, we detected low-level viral genomes in several plasma samples and this is consistent with the literature of JCPyV reactivation following natalizumab administration [7]. We also detected viral genomes in one plasma sample from a patient treated with teriflunomide. Because patient samples were limited, a larger, more comprehensive study would be required to draw conclusions from the comparison of natalizumab- versus teriflunomide-treated patients. The trend, however, points to increased viral genomes in natalizumab-treated patients versus patients who did not receive natalizumab. Risk stratification based on ddPCR or quantitative PCR would clearly be more sensitive and accurate than relying on the current standard of measuring antibody indices [31,33,34]. None of the CSF samples from non-PML patients were positive. We could not obtain CSF from natalizumab-treated patients for direct comparison to CSF from teriflunomide-treated patients.

## 4. Materials and Methods

### 4.1. Cells, Media, and Virus

Human CPEpic-L were obtained from ScienCell Research Labs (Carlsbad, CA, USA) and cultured in cell line-specific complete media, as indicated by the manufacturer (ScienCell), in a humidified incubator at 37 °C with 5% CO_2_. CPEpic-L were immortalized as previously described [13,14]. Plasmids for immortalization—pLV-hTERT IRES hygro (85140), pCMVdr8.9 (8455), and pVSV-G (8454)—were purchased from Addgene (Watertown, MA, USA). Primary human astrocytes were obtained from ScienCell Research Labs and cultured in cell line-specific complete media, as indicated by the manufacturer (ScienCell, Carlsbad, CA, USA), in a humidified incubator at 37 °C with 5% CO_2_. In brief, 1 × 10^6^ cells at passage 1 were plated on poly-l-lysine coated flasks in complete media. Media was replaced every other day until the culture reached 90% confluency, at which point cells were seeded to multi-well dishes as appropriate for each experiment. The Mad1/SVEdelta strain of JCPyV was propagated in SVGA cells and purified using cesium chloride as previously described [35].

### 4.2. Proliferation and Toxicity

To determine the cytostatic effect of teriflunomide, cells were treated, infected with cesium chloride-purified JCPyV, and maintained in teriflunomide or DMSO for 5 days as described above. On Day 5, cells were collected using Cellstripper (Corning Inc., Corning, NY, USA), stained with propidium iodide (Sigma-Aldrich, St. Louis, MO, USA), and analyzed in triplicate by flow cytometry for total, live, and dead cell counts (BD FACSCanto II, BD Biosciences, Franklin Lakes, NJ, USA). Volume-matched vehicle controls were run for each sample. To determine toxicity, cells were plated at a density of 10,000 cells/cm^2^ in 96-well plates and pretreated with either teriflunomide (0.1 μM to 2 mM) or volume-matched DMSO vehicle control. Cells were pretreated for 1 h with teriflunomide or DMSO, infected, and maintained with teriflunomide or vehicle control for the duration of the experiment: 5 days for initial infection or 15 days for long-term infection. Viability was determined by visual inspection of the cells followed by the addition of 20 μL of a tetrazolium compound (CellTiter 96 Aqueous One Solution Cell Proliferation Assay [MTS] [Promega, Madison, WI, USA]). The plate was incubated for 2 h at 37 °C and absorbance was read at 450 nm using a plate reader (Glomax, Promega).

### 4.3. Infections

For initial infection experiments, cells were plated at 10,000 cells/cm^2^ in poly-l-lysine–coated 96-well plates and pretreated with teriflunomide or DMSO in serum-free media for 1 h at 37 °C. Following treatment, cells were challenged with cesium chloride–purified JCPyV for an additional 1 h at 37 °C. Virus-containing media was then aspirated and infected cultures were maintained in the presence of teriflunomide or DMSO across a range of concentrations. Cells were fixed at 5 days post infection using ice-cold methanol. For growth assay infections, cells were infected as described above. Twenty-four hours following infection, media containing teriflunomide or vehicle control (DMSO) was added to infected cultures. Samples in triplicate were fixed in ice-cold methanol every 5 days post infection for a total of 15 days. For rescue experiments, cultures were treated using teriflunomide at the highest tolerable dose for each cell type followed by uridine or orotic acid as indicated (Figure 4 and Figure 5). Cultures were infected with cesium chloride-purified JCPyV for an additional 1 h at 37 °C, and maintained post infection with and without treatment, as indicated.

### 4.4. Indirect Immunofluorescence

Infected cultures were fixed in ice-cold methanol for 20 min and allowed to rehydrate for 15 min in phosphate-buffered saline (PBS), followed by incubation with VP1-specific antibody PAB597 in PBS at 37 °C for 1 h. Following primary antibody incubation, cells were washed with PBS and incubated with a goat anti-mouse Alexa fluor 488 conjugated antibodies in PBS at 37 °C for 1 h. Secondary antibody was washed out and cells were counterstained with DAPI (4′,6-diamidino-2-phenylindole) in PBS for 5 min at room temperature. Cells were analyzed for nuclear VP1 staining and total cell number under a 20× objective lens using a Ti2-E fluorescent microscope (Nikon Inc., Chicago, IL, USA). Cell count analysis was performed using Elements High Content imaging software (Nikon).

### 4.5. Patient Samples

An MS patient library was purchased from Innovative Research (Novi, MI, USA). A cohort of teriflunomide-treated, natalizumab-treated, or nonimmunomodulated relapsing-remitting patient plasma, serum, and CSF from anonymized donors was chosen. Patient samples were matched to the best of our ability for subtype diagnosis (RRMS), age, sex, and the absence of potentially confounding conditions such as chemotherapeutic medication. Pooled healthy donor plasma, serum, and CSF were also used as negative controls and to generate spike-in positive controls. Each pooled plasma sample represents approximately two healthy donors, each pooled serum sample represents ≥25 healthy donors, and each pooled CSF sample represents three to five healthy donors. Plasma and CSF from two patients with known PML were provided by Northwestern University. Informed consent was obtained from all participants in the study. Documentation is retained by the admitting hospitals.

### 4.6. Generation of Control Samples

Negative controls of plasma, serum, and CSF were purchased as a pooled healthy donor sample from Innovative Research. Positive controls were generated for each biofluid by spiking 250 µL of healthy donor fluid with 5 μL of infectious EV and either used for total sample analysis or processed for EV. Infectious EV used for spike-in controls were concentrated 7 days post infection from SVGA tissue cultures as previously described [32]. A 250-µL sample of each biofluid was also processed without the addition of infectious EV as the negative control and either used for total sample analysis or processed for EV. Additional controls included a no-template control (NTC) and an extraction blank; sterile PBS was run through the isolation protocol identically to the patient samples to ensure the integrity of the extraction kit. To verify the accuracy of the probes, a standard curve was generated using serial dilutions of digested Mad1-pBR322 DNA.

### 4.7. Extracellular Vesicles

In a dedicated biosafety cabinet, EV were isolated from patient biofluid using size-exclusion chromatography followed by ultrafiltration to concentrate EV. In a dedicated clean tissue culture hood, samples were thawed on ice. Two hundred fifty μL of serum, plasma, or CSF was precleared of debris by centrifugation at 10,000× *g* for 20 min at room temperature. Single-use size-exclusion chromatography mini columns (qEV-single, Izon) were equilibrated to room temperature and washed twice with two column volumes of 0.22 μM fresh filtered 1× PBS. Following column washes, cleared biofluids were applied directly to the column matrix as recommended by the manufacturer. The initial 1-mL void volume was discarded and 10,200-μL fractions were collected using an Izon qEV fraction collector (Izon Science, Christchurch, NZ). Fractions F1–F5 were pooled and concentrated by ultrafiltration using an Amicon Ultra-4 centrifugal filter (30,000 nominal molecular weight limit) by centrifugation for 10 min at 4000× *g* (Merck-Millipore, Burlington, MA, USA). Columns were washed with sterile, particle-free water immediately prior to EV concentration to remove residual column glycerol. The concentrated EV sample was removed from the filter unit and immediately processed for DNA extraction and characterization. Exoview analysis and characterization was contracted to NanoView Biosciences (Nanoview Biosciences, Boston, MA, USA).

### 4.8. DNA Extraction

In a dedicated biosafety cabinet, 20 μL of each patient-derived EV sample was first treated with DNase I (New England BioLabs, Ipswich, MA, USA) to remove unencapsidated DNA. Samples were incubated at 37 °C for 30 min followed by inactivation at 75 °C for 10 min. DNase-treated EV samples were processed for DNA extraction using the Qiagen Blood and Tissue kit. Samples were eluted in 100 μL sterile molecular-grade water. Total fluid samples were processed with 50 μL initial input using the Qiagen Blood and Tissue kit and eluted similarly in 100 μL sterile molecular-grade water (Qiagen, Germantown, MD, USA). Using reduced sample input for total fluid was necessary due to the low sample volumes available.

### 4.9. Droplet Digital PCR

In preparation for ddPCR, samples were digested overnight at 4 °C with BamHI-HF (New England Biolabs). PrimerTime Assay primer/probe sets were purchased from IDT (Newark, NJ, USA). Sequences are as follows: JCPyV T-antigen primer 1: GGCAATGCACTGAAGGATTAG; JCPyV T-antigen primer 2: GTTCAGAGGTTGGTTGTGATTT; JCPyV T-antigen probe: /56-FAM/TTGCAAGGA/ZEN/ATGGCCTAACTGTGC/3IABkFQ/; JCPyV VP1 primer 1: AGGGACATGCTTCCTTGTTAC; JCPyV VP1 primer 2: CAGCCTCCCACATGAGTATATTT; JCPyV VP1 probe: /5HEX/TGTGGCCAG/ZEN/AATTCCACTACCCAA/3IABkFQ/. A 2.2-μL input of digested sample was used per 22 μL ddPCR reaction. Samples and controls were run in triplicate. Droplets were generated using a BioRad Automated Droplet Generator (Bio-rad Laboratories, Hercules, CA, USA). Cycling conditions for PCR were as follows: 10 min at 95 °C; 40 cycles at 94 °C for 30 s and 60 °C for 60 s; one cycle at 98 °C for 10 min. Following thermocycling, droplets were stabilized for either 4 h at 12 °C or overnight at 4 °C. Stabilized plates were equilibrated to room temperature and analyzed using a BioRad QX200 Droplet Reader (Bio-rad Laboratories, Herucles, CA, USA). Data were processed using BioRad QuantaSoft software (Bio-rad Laboratories, Hercules, CA, USA). To be considered positive for JCPyV, samples must have had two or more fluorescent droplets and droplets must have been at the expected amplitude as determined by the positive controls for each probe set. In addition, there must have been consensus between probe sets. In the context of this assay, two droplets is equivalent to approximately 120 viral copies per mL.

## 5. Statistical Analysis

A Student’s *t*-test was used to calculate the p-value of each experiment using Microsoft Excel. A *p*-value of less than 0.05 was considered significant and is indicated in figures by *. Where there is no significant change, *p* > 0.05, the figure legend indicates NS for no significance. Infection is reported on the median value of VP1+ cells. Experiments were performed in triplicate. The interquartile range (IQR) to describe the variability for each sample was calculated in Microsoft Excel and is represented by error bars in Figure 2, Figure 3, and Figure 4, as stated in the figure legends. The standard deviation is represented by error bars in Figure 1, Figure 5, and Figure 6 and was calculated using Microsoft Excel. The standard error was used in Figure 8 and Figure 9 and was calculated in the QuantaSoft software.

## Figures and Tables

**Table 1:** JCPyV was detected in plasma and serum of one nonimmunomodulated patient and in plasma from three of seven natalizumab-treated patients in the EV fraction; six of seven patients were positive for JCPyV when the total sample was analyzed. JCPyV was not detected in any RRMS CSF samples and was not detected in plasma- or serum-derived EV from teriflunomide-treated patients; one plasma and one serum as total sample were JCPyV positive. PML control patients were positive for JCPyV in plasma and CSF, both in total and EV fractions. N/A indicates the sample was not available for testing. HIV = human immunodeficiency virus; RRMS = relapsing-remitting multiple sclerosis.

	JCPyV Status					
	CSF (+/−)		Plasma (+/−)		Serum (+/−)	
Patient Group	Total	EV	Total	EV	Total	EV
RRMS, teriflunomide	0/7	0/7	1/1	0/2	1/5	0/6
RRMS, natalizumab	N/A	N/A	6/7	3/4	0/7	0/7
Nonimmunomodulated	0/2	0/2	1/1	1/1	1/1	1/1
HIV-PML	2/0	2/0	2/0	2/0	N/A	N/A

## Data Availability

Data are contained within the article and its Supplementary Materials.

## Supplementary Materials

The following are available online at https://www.mdpi.com/article/10.3390/ijms22189809/s1.

## Previous Presentation

Some of the data included in this manuscript have been presented at the 35th Congress of the European Committee for Treatment and Research in Multiple Sclerosis (ECTRIMS), 11–13 September 2019, Stockholm, Sweden, and the 72nd Virtual Annual Meeting of the American Academy of Neurology (AAN), 25 April to 1 May 2020.
