# Supplementary material for: Teriflunomide Inhibits JCPyV Infection and Spread in Glial Cells and Choroid Plexus Epithelial Cells
Source: Int J Mol Sci. 2021 Sep 10;22(18):9809. doi: 10.3390/ijms22189809 (PMC8468119; doi:10.3390/ijms22189809)
Supplement: Supplementary file 1 [file ijms-22-09809-s001.zip › ijms-1323523-supplementary.pdf]

Table S1. Patient samples were analyzed by ddPCR for the presence of JCPyV DNA in both EV and total sample. Results are stratified by matched patients and unmatched patients. The absolute copy number for all JCPyV-positive patients is reported as detected genome copies/mL. Samples that were below the threshold or that did not have consensus between probes are reported as JCPyV negative (NEG). N/A indicates the sample was not available for analysis. NIM = nonimmunomodulated.

Diagnosis	Treatment	Sample ID	Type	Target	CSF	Plasma	Serum
**Matched patients**							
RRMS	Teriflunomide	537695/96/97	Total	T-ag	NEG	397	NEG
				VP1	NEG	126	NEG
			EV	T-ag	NEG	NEG	NEG
				VP1	NEG	NEG	NEG
RRMS	Teriflunomide	603099/00/01	Total	T-ag	NEG	200	NEG
				VP1	NEG	146	NEG
			EV	T-ag	NEG	NEG	NEG
				VP1	NEG	NEG	NEG
RRMS	NIM	613029/30/31	Total	T-ag	NEG	263	250
				VP1	NEG	290	280
			EV	T-ag	NEG	NEG	120
				VP1	NEG	NEG	725
RRMS	NIM	631005/06/07	Total	T-ag	NEG	NEG	320
				VP1	NEG	NEG	340
			EV	T-ag	NEG	275	5500
				VP1	NEG	1000	4830
RRMS	Teriflunomide	514866/68	Total	T-ag	NEG	N/A	NEG

Diagnosis	Treatment	Sample ID	Type	Target	CSF	Plasma	Serum
				VP1	NEG	N/A	NEG
			EV	T-ag	145	N/A	415
				VP1	305	N/A	120
RRMS	Teriflunomide	505961/63	Total	T-ag	NEG	N/A	120
				VP1	NEG	N/A	140
			EV	T-ag	NEG	N/A	185
				VP1	NEG	N/A	675
RRMS	Teriflunomide	506459/91	Total	T-ag	NEG	N/A	NEG
				VP1	NEG	N/A	NEG
			EV	T-ag	NEG	N/A	NEG
				VP1	NEG	N/A	NEG
RRMS	Teriflunomide	50746/58	Total	T-ag	NEG	N/A	NEG
				VP1	NEG	N/A	NEG
			EV	T-ag	NEG	N/A	NEG
				VP1	NEG	N/A	NEG
RRMS	Natalizumab	616760/62	Total	T-ag	N/A	333	NEG
				VP1	N/A	273	NEG
			EV	T-ag	N/A	240	120
				VP1	N/A	345	345
RRMS	Natalizumab	613050/52	Total	T-ag	N/A	423	NEG
				VP1	N/A	243	NEG

Diagnosis	Treatment	Sample ID	Type	Target	CSF	Plasma	Serum
			EV	T-ag	N/A	120	NEG
				VP1	N/A	675	NEG
PML	Unknown	1015193	Total	T-ag	4900	4500	N/A
				VP1	5000	4300	N/A
			EV	T-ag	640	300	N/A
				VP1	670	243	N/A
PML	Unknown	1518105	Total	T-ag	8600	380	N/A
				VP1	8500	810	N/A
			EV	T-ag	273	223	N/A
				VP1	500	300	N/A
**Unmatched patients**							
RRMS	Teriflunomide	510050	Total	T-ag	NEG	N/A	N/A
				VP1	NEG	N/A	N/A
			EV	T-ag	NEG	N/A	N/A
				VP1	NEG	N/A	N/A
RRMS	Natalizumab	227994	Total	T-ag	N/A	400	N/A
				VP1	N/A	166	N/A
			EV	T-ag	N/A	NEG	N/A
				VP1	N/A	NEG	N/A
RRMS	Natalizumab	304340	Total	T-ag	N/A	NEG	N/A
				VP1	N/A	NEG	N/A

Diagnosis	Treatment	Sample ID	Type	Target	CSF	Plasma	Serum
			EV	T-ag	N/A	385	N/A
				VP1	N/A	575	N/A
RRMS	Natalizumab	611700	Total	T-ag	N/A	NEG	N/A
				VP1	N/A	NEG	N/A
			EV	T-ag	N/A	NEG	N/A
				VP1	N/A	NEG	N/A
RRMS	Natalizumab	211670	Total	T-ag	N/A	200	N/A
				VP1	N/A	207	N/A
			EV	T-ag	N/A	350	N/A
				VP1	N/A	830	N/A
RRMS	Natalizumab	216647	Total	T-ag	N/A	370	N/A
				VP1	N/A	620	N/A
			EV	T-ag	N/A	1100	N/A
				VP1	N/A	207	N/A
RRMS	Natalizumab	578000	Total	T-ag	N/A	N/A	NEG
				VP1	N/A	N/A	NEG
			EV	T-ag	N/A	N/A	NEG
				VP1	N/A	N/A	NEG
RRMS	Natalizumab	614111	Total	T-ag	N/A	N/A	NEG
				VP1	N/A	N/A	NEG

Diagnosis	Treatment	Sample ID	Type	Target	CSF	Plasma	Serum
			EV	T-ag	N/A	N/A	NEG
				VP1	N/A	N/A	NEG
RRMS	Natalizumab	458688	Total	T-ag	N/A	N/A	NEG
				VP1	N/A	N/A	NEG
			EV	T-ag	N/A	N/A	NEG
				VP1	N/A	N/A	NEG
RRMS	Natalizumab	644018	Total	T-ag	N/A	N/A	NEG
				VP1	N/A	N/A	NEG
			EV	T-ag	N/A	N/A	NEG
				VP1	N/A	N/A	NEG
RRMS	Natalizumab	607678	Total	T-ag	N/A	N/A	NEG
				VP1	N/A	N/A	NEG
			EV	T-ag	N/A	N/A	NEG
				VP1	N/A	N/A	NEG
